# Supplementary material for: Mimicking Sampson’s Retrograde Menstrual Theory in Rats: A New Rat Model for Ongoing Endometriosis-Associated Pain
Source: Int J Mol Sci. 2020 Mar 27;21(7):2326. doi: 10.3390/ijms21072326 (PMC7177935; doi:10.3390/ijms21072326)
Supplement: Supplementary file 1 [file ijms-21-02326-s001.pdf]

Supplementary

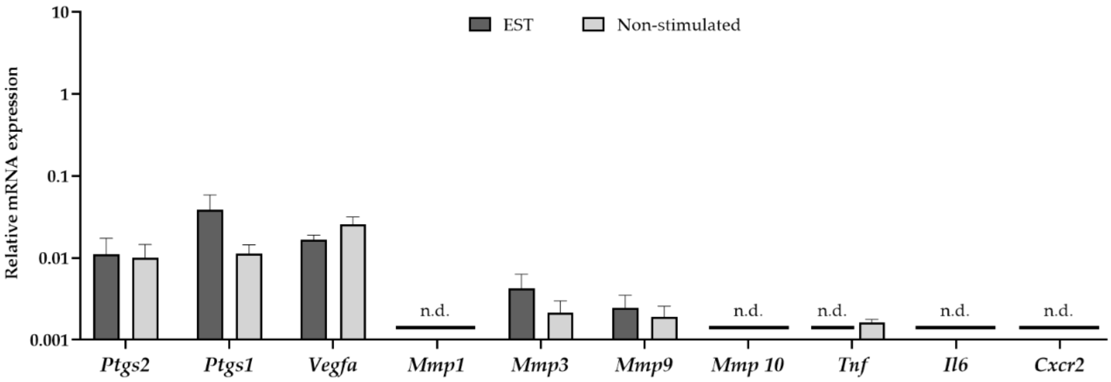

Figure S1. mRNA expression of menstrual genes in the naïve endometrial tissue and non-stimulated MRM tissue. cDNA was synthesized from endometrial tissue, which was either naïve endometrium from the pro-estrus phase (EST; n = 3) or endometrium that underwent MRM and kept the P4 pellet but did not receive the decidualization stimulus (non-stimulated; n = 3). Messenger RNA levels were quantified to the geometric mean of housekeeping genes *β-actine (Actb), Glyceraldehyde 3-phosphate dehydrogenase (Gapdh)* and *TATA-binding protein (TBP)*. *Mmp1, Mmp10, Tnf, Il6* and *Cxcr2* were around (30 < Cq < 35) or below (Cq > 35) the detection limit, and were indicated as not determined (n.d.). Data are presented as mean ± SD. Statistically significant changes in mRNA expression were assessed using the two-way ANOVA statistical test with Bonferroni correction.

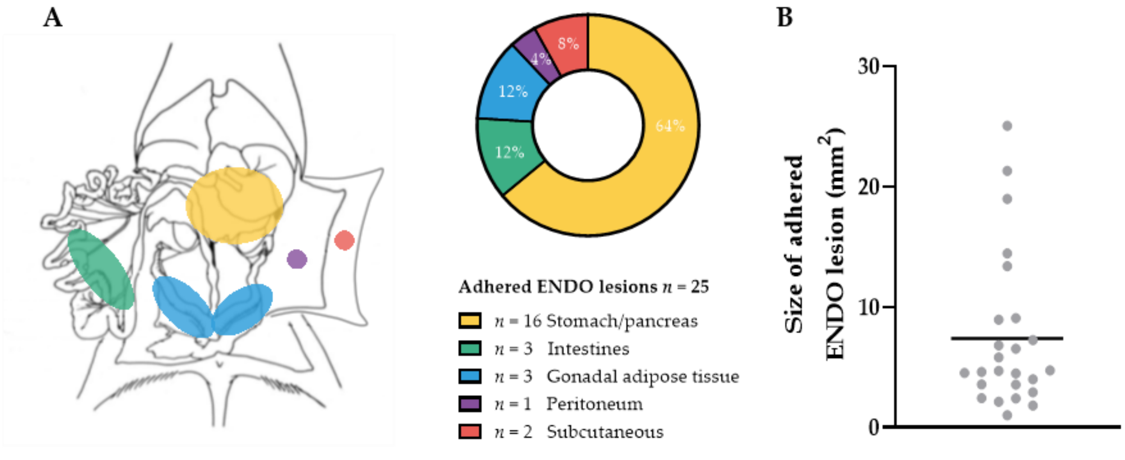

Figure S2. Anatomical location of adhered endometriosis lesions. (**A**) In general, endometriosis lesions adhere in the stomach and pancreatic region (64% of adhered lesions). Furthermore, lesions are often found on the intestines/intestinal connective and gonadal adipose tissue (both 12% of adhered lesions). In rare cases, adhered lesions are observed on the peritoneum or subcutaneously (4% and 8%, respectively) (n = 25 from collected from 18 recipient rats). (**B**) The size of adhered ENDO lesions. Outline of rodent anatomy is adapted from Jackson Laboratories [38] and Dodds et al. [28].

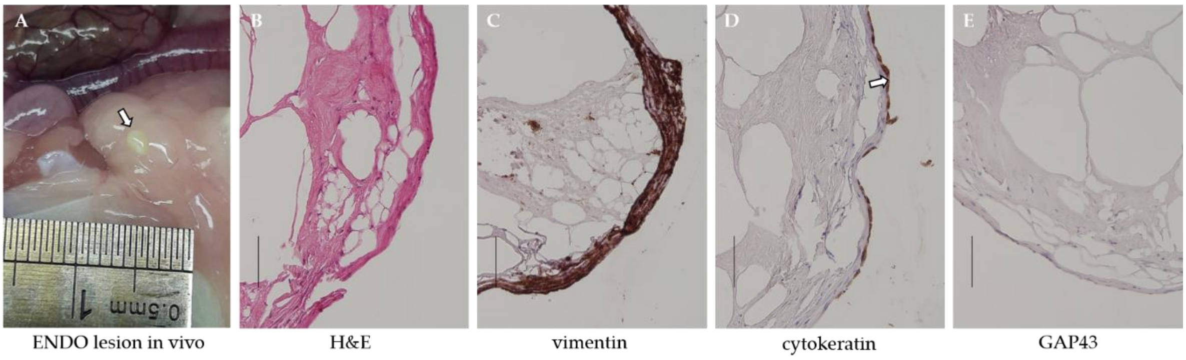

Figure S3. The cellular morphology of non-adhered tissue. (**A**) Non-adhered tissue (white arrow). (**B**) H&E staining shows the general morphology, indicating the absence of blood vessels. (**C**) The stromal cells of the lesion can be identified using vimentin staining. (**D**) Endometrial epithelial cells are identified by cytokeratin (white arrow). (**E**) However, no nerve fibers are observed in the non-adhered tissue as indicated by a negative GAP43 staining. Scale 100 μm.

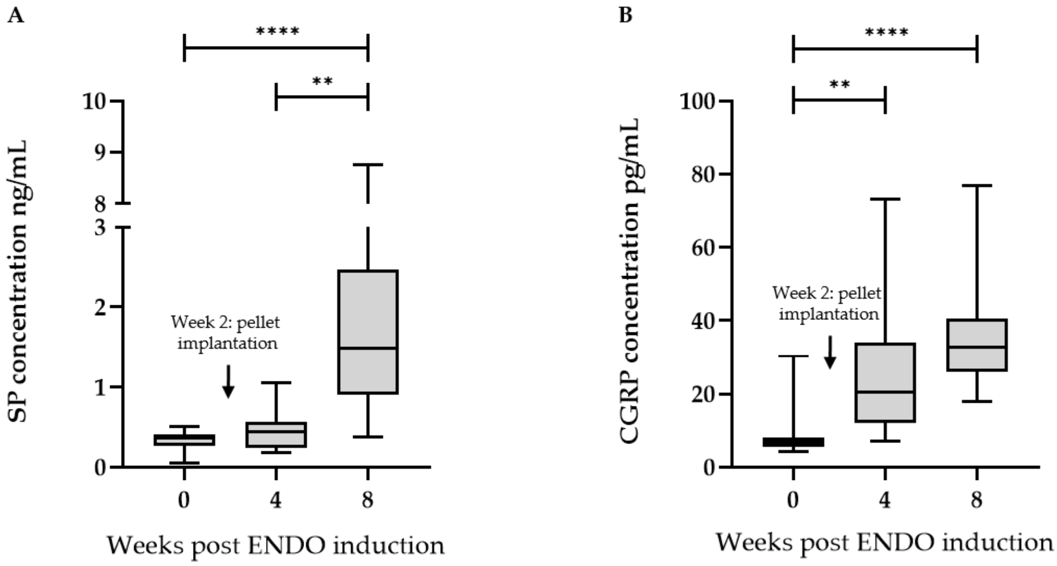

Figure S4. Neuropeptide supplementation of SP and/or CGRP with an osmotic pump increases the plasma concentration. (**A**) SP plasma concentrations are significantly elevated 6 weeks after pellet implantation (*i.e.*, 8 weeks post ENDO induction) (n = 35 recipient rats, measured over three time points). (**B**) Likewise, plasma levels for CGRP were significantly increased 2 weeks after pellet implantation (*i.e.*, 4 weeks post ENDO induction) and remained elevated until week 8 (n = 14 recipient rats, measured over three time points). Statistical differences were detected using a Kruskal-Wallis test with Dunn's correction.

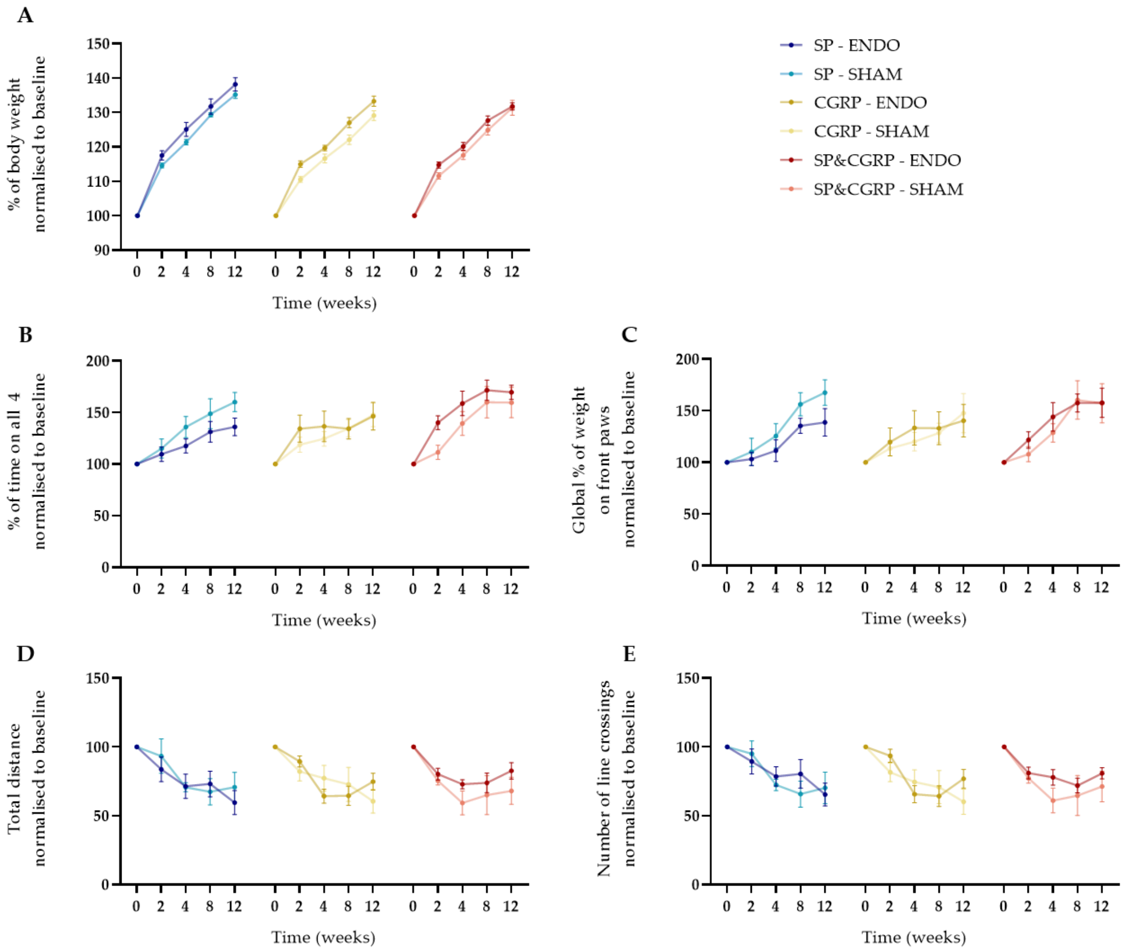

Figure S5. Neuropeptide supplementation does not affect ongoing endometriosis-associated pain. (**A**) No significant difference in body weight was observed between ENDO and SHAM animals after SP, CGRP or SP&CGRP neuropeptide supplementation. (**B**,**C**) When assessing the ADWB parameters % of time on all 4 or global % of weight on front paws, neither SP, CGRP nor SP&CGRP caused a significant difference between the ENDO and SHAM groups (**D**,**E**) Likewise, no difference was observed in total distance or number of line crossings during the open field assay. All groups $n = 10$. Data presented as mean ± SEM. Statistical differences were assessed using a two-way ANOVA with Bonferroni correction.

Table S1. Optimization of the menstruating rat model

Optimalisation High E2 Injection

	0.1 μg E2				0.5 μg E2					1 μg E2				2 μg E2			
Day 7	D	D	D	D	D	D	D	D	D	D	D	D	D	D	D	D	D
Day 8	D	D	D	D	D	D	D	D	P	D	D	P	D	E	D	P	P
Day 9	P	D	P	D	P	P	P	P	P	P	P	P	P	E	E	E	E
Day 10	E	P	P	P	E	E	E	E	E	E	E	E	E	E	E	E	E

Optimalisation Low E2 Injection & P4 Pellet

	0.005 μg E2 + P4 pellet					0.01 μg E2 + P4 pellet				
Day 7	D	D	D	D	D	D	D	D	D	D
Day 8	D	D	D	D	D	D	D	D	D	D
Day 9	P	P	P	P	P	P	P	P	P	P
Day 10	E	E	E	E	E	E	E	E	E	E
Day 11	D	D	D	M	M	D	D	D	M	M
Day 12	D	D	D	D	D	D	D	D	D	D
Day 13	D	D	D	D	D	D	D	D	D	D
Day 14	D	D	D	D	D	D	D	D	D	D
Day 15	D	D	D	D	D	D	D	D	D	D
Day 16	D	D	D	D	D	D	D	D	D	D

Optimalisation Day Of Decidualization Stimulus

	Day 14						Day 15				
Day 7	D	D	D	D	D	Day 7	D	D	D	D	D
Day 8	D	D	D	D	D	Day 8	D	D	D	P	D
Day 9	P/E	P	P	E	P	Day 9	P	P	P	E	P
Day 10	E	E	E	E	E	Day 10	E	E	E	E	E
Day 11	n.d.	n.d.	n.d.	n.d.	n.d.	Day 11	n.d.	n.d.	n.d.	n.d.	n.d.
Day 12	D	D	D	D	M	Day 12	D	D	D	M	M
Day 13	D	D	D	D	D	Day 13	D	D	D	D	D
Day 14	D	D	D	D	D	Day 14	D	D	D	D	D
Day 15	n.d.	n.d.	n.d.	n.d.	n.d.	Day 15	D	D	D	D	D
Day 16	n.d.	n.d.	n.d.	n.d.	n.d.	Day 16	n.d.	n.d.	n.d.	n.d.	n.d.
Day 17	n.d.	n.d.	n.d.	n.d.	n.d.	Day 17	n.d.	n.d.	n.d.	n.d.	n.d.
Day 18	n.d.	n.d.	n.d.	n.d.	n.d.	Day 18	n.d.	n.d.	n.d.	n.d.	n.d.
Day 19	n.d.	n.d.	n.d.	n.d.	n.d.	Day 19	n.d.	n.d.	n.d.	n.d.	n.d.
	+	+	+	+	+		+	+	+	+	+

E2 estradiol-17β; P4 progesterone; D di-estrus; P pro-estrus; E estrus; M met-estrus

Table S2. Histological assessment of adhered rat endometriosis lesions

Lesion n°	Protocol	H&E Presence Blood Vessels	Vimentin	Cytokeratin	GAP43
Recipient 2 L2	Regular	+	+	+	+
Recipient 2 L3	Regular	+	+	-	+
Recipient 4 L1	Regular	+	+	+	-
Recipient 6 L1	Regular	+	+	+	-
Recipient 10 L1	Regular	+	-	+	+
Recipient 11 L1	Regular	+	+	+	+
Recipient 11 L2	Regular	+	+	+	+
Recipient 11 L3	Regular	+	+	+	+
Recipient 11 L4	Regular	+	+	+	-
Recipient 12 L1	Regular	+	+	+	+
Recipient 16 L1	Regular	+	+	+	+
Recipient 17 L1	Regular	+	+	+	+
Recipient 17 L2	Regular	+	+	+	+
Recipient 18 L2	Regular	+	+	+	-
Recipient 31 L3	Regular	+	+	+	+
Recipient 34 L1	Regular	+	+	+	-
Recipient 34 L2	Regular	+	+	+	+
Recipient 35 L1	Regular	+	+	+	+
Recipient 38 L1	Regular	+	+	+	-
Recipient 38 L2	Regular	n.d.	n.d.	n.d.	n.d.
Recipient 61 L1	Regular	+	+	+	+
Recipient 62 L1	Regular	+	+	+	+
Recipient 64 L1	Regular	+	+	+	+
Recipient 66 L1	Regular	+	+	+	-
Recipient 69 L1	Regular	+	+	+	+
		**100%**	**96%**	**96%**	**71%**
Recipient 72 L1	SP	+	+	+	+
Recipient 74 L1	SP	+	+	+	-
Recipient 76 L1	SP	-	+	-	-
Recipient 77 L1	SP	n.d.	n.d.	n.d.	n.d.
Recipient 79 L1	SP	+	+	+	-
		**75%**	**100%**	**75%**	**25%**
Recipient 91 L1	CGRP	+	+	+	+
Recipient 92 L1	CGRP	+	+	+	-
Recipient 93 L1	CGRP	+	+	+	+
Recipient 93 L2	CGRP	n.d.	n.d.	n.d.	n.d.
Recipient 98 L1	CGRP	+	+	+	+
Recipient 99 L1	CGRP	+	+	+	+
		**100%**	**100%**	**100%**	**80%**
Recipient 111 L1	SP&CGRP	+	+	-	+
Recipient 112 L1	SP&CGRP	+	+	+	+
Recipient 118 L1	SP&CGRP	n.d.	n.d.	n.d.	n.d.
		**100%**	**100%**	**50%**	**100%**

Table S3. List of used Taqman genes (Applied Biosystems).

Gene Name	Assay ID	RefSeq ID	Exon Boundary	Assay Location	Amplicon Length
Actb	Rn00667869_m1	NM_0341144.3	4–5	881	91
Gapdh	Rn01775763_g1	NM_017008.4	8–8	1153	174
Tbp	Rn01455646_m1	NM_001004198.1	4–5	791	75
Ptgs2	Rn01483828_m1	NM_017232.3	3–4	312	112
Ptgs1	Rn00566881_m1	NM_017043.4	9–10	1364	85
Vegfa	Rn01511602_m1	NM_001110333.2	3–4	1328	95
Ednra	Rn00561137_m1	NM_012550.2	2–3	715	118
Mmp1	Rn01486634_m1	NM_001134530.1	7–8	1062	88
Mmp3	Rn00591740_m1	/	/	1379	67
Mmp9	Rn00579162_m1	NM_031055.1	12–13	2016	72
Mmp10	Rn00591678_m1	NM_133514.1	6–7	968	79
Tnf	Rn99999017_m1	NM_012675.3	2–3	385	108
IL6	Rn01410330_m1	NM_012589.2	3–4	383	121
Cxcr2	Rn02130551_s1	NM_017183.1	2–2	1282	122
